# Train the trainers: replicating the message of hand hygiene promotion through the training of national experts, preliminary results

**DOI:** 10.1186/2047-2994-4-S1-P285

**Published:** 2015-06-16

**Authors:** F Bellissimo-Rodrigues, A Agostinho, D Pittet

**Affiliations:** 1 Infection Control Programme, University of Geneva Hospitals, Geneva, Switzerland; 2 Collaborating Centre on Patient Safety, World Health Organization (WHO), Geneva, Switzerland

## Introduction

Despite being the most effective preventive measure against health-care associated infections (HAI), hand hygiene (HH) practices are still suboptimal all around the world, with compliance among healthcare workers generally falling below 50% of the opportunities to perform it. One of the potential strategies for HH promotion at national level is to “Train the Trainers”, i.e., to train well-recognized national experts on the implementation of the World Health Organization (WHO) Multimodal Strategy for HH Improvement.

## Objectives

We aimed to describe the realization of a “Train the Trainers” course, and to evaluate its immediate impact on the participant experts knowledge about HH.

## Methods

This is a quasi-experimental study based on a questionnaire, applied to all participants immediately before and after the course, which took place in Rio de Janeiro, Brazil, from February 23 to 25, 2015. The questionnaire consisted of 15 multiple affirmatives that should be judged as true or false by responders. Questions addressed HH issues around the use of alcohol-based handrub (n=5), soap and water (n=5), and surgical hand preparation (n=5). McNemar’s test was used to compare the results before and after the training.

## Results

A total of 33 infection control practitioners attended the course and completed both questionnaires. Overall, the rate of correct answers was 77.0% before and 89.7% after the course (p<0.001). Regarding handrubbing with alcohol, the rate of correct answers was 88.2% before and 92.1% after the course (p<0.001). Regarding handwashing, the rate of correct answers was 76.4% before and 92.1% after the course (p=0.029). Regarding surgical hand preparation, the rate of correct answers was 70.3% before and 84.9% after the course (p<0.001).

## Conclusion

An intensive 3 days course about the WHO Multimodal Strategy for HH Improvement proved to be effective in enhancing the corresponding knowledge of participant experts. Further studies should access the training program effectiveness to improve HH practices at national level.

## Disclosure of interest

None declared.

